# Penile Intraepithelial Neoplasia in an Asian Male: A Case Report

**DOI:** 10.7759/cureus.69412

**Published:** 2024-09-14

**Authors:** Deepanshu Sharma, Imran Ali Khan, Anup A Zade, Sai Goutham Rekavari, Shruthi Bikkumalla

**Affiliations:** 1 Department of General Surgery, Jawaharlal Nehru Medical College, Datta Meghe Institute of Higher Education and Research, Wardha, IND

**Keywords:** glans resurfacing, neoplasia, penile intraepithelial neoplasia, penile reconstruct, squamous cell carcinoma

## Abstract

Penile intraepithelial neoplasia is a rare presentation in Asian males. It is usually observed as small lesions in the penis. It primarily influences the penetrative sexual intercourse ability and urination. Major risk factors associated are smoking, human papillomavirus infection, phimosis, tobacco consumption, and poor hygiene practices. Prompt diagnosis and treatment can lead to good prognostic outcomes. A 70-year-old male presented with penile swelling, weight loss, and burning micturition. The lesion on initial presentation looked malignant clinically due to the abnormal size of the lesion. This was later found to be premalignant/benign on histopathology. The lesion was timely treated saving the patient from losing organ functionality and from the terrifying effects of chemotherapy.

## Introduction

Penile intraepithelial neoplasia is a rare precancerous condition affecting the outer skin layer of the penis, primarily the glans and foreskin, majorly affecting urination and penetrative sexual intercourse [1]. It is characterized by abnormal cell changes that can potentially progress to invasive squamous cell carcinoma if left untreated [2]. Penile intraepithelial neoplasia is often classified into subtypes, including undifferentiated and differentiated forms, with the undifferentiated type being more common [3]. It is considered a precursor to penile cancer and has shown a slight increase in incidence from 0.85/100,000 person-years in 1997-1998 to 1.13/100,000 person-years in 2017-2018 [4]. It is rare but can sometimes be observed in Asian males between 60 and 70 years [5]. It typically presents as single or multiple lesions that may appear as red plaques on the glans or inner foreskin with different textures [2]. Symptoms may include painful urination, discharge, and difficulty retracting the foreskin. Treatment options vary based on the severity and recurrence of the lesions. Surgical intervention, particularly circumcision, is often the primary treatment, as topical treatments alone have shown limited effectiveness. Mohs micrographic surgery is also considered effective for severe cases [1,6]. This is a case of a 70-year-old male with a primary complaint of penile swelling for six months. The diagnosis was later confirmed to be penile intraepithelial neoplasia grade 3 on histopathological analysis.

## Case presentation

**Figure 1:**
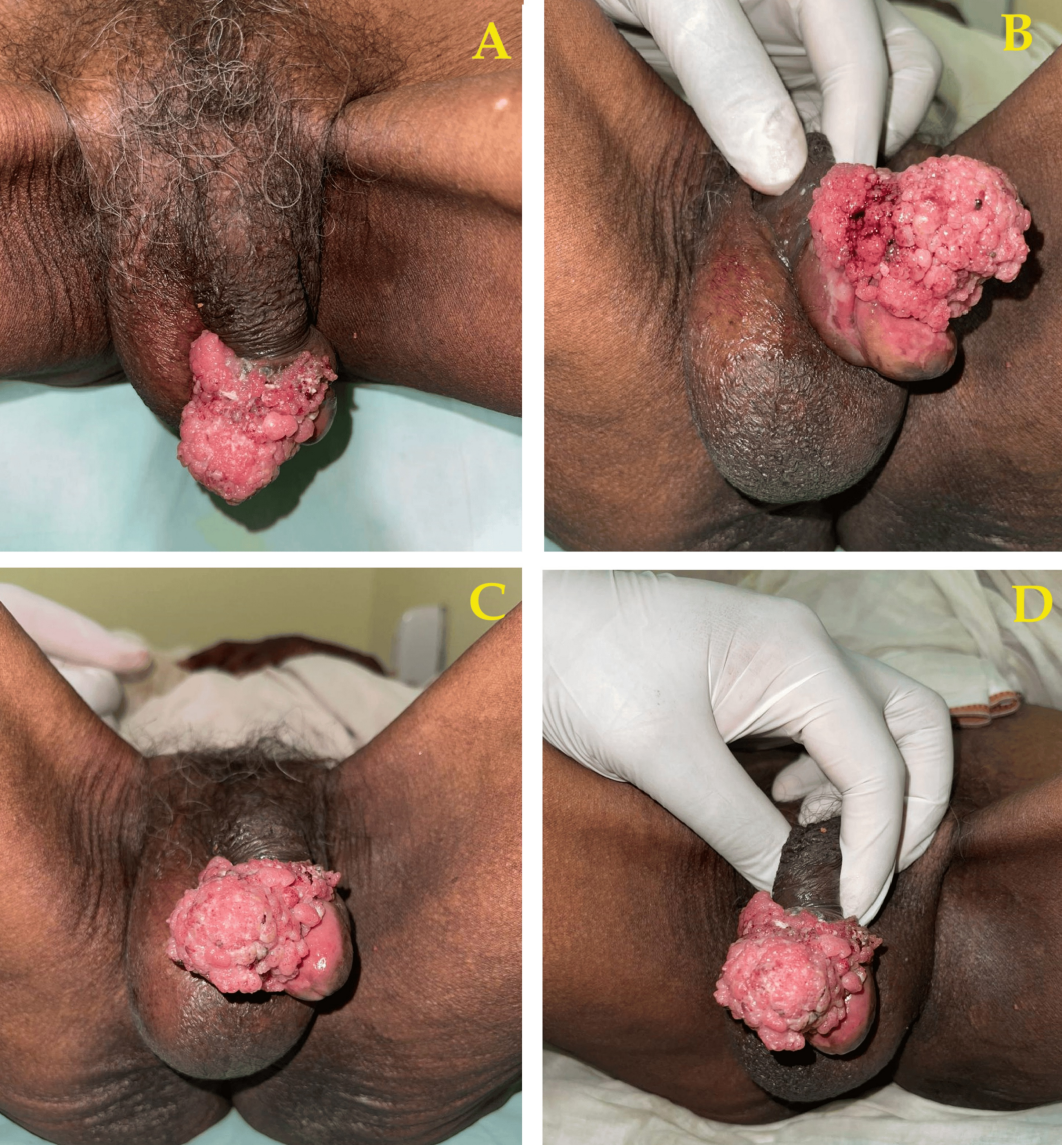
Physical presentation of the penile swelling. (A,B) The extent of growth around the penile region. (C,D) The extent of the glans of the penis

A male patient of 70 years presented with a complaint of ulceroproliferative growth over the penile region for the past six months. The patient did not have any other medical conditions before six months. The swelling onset was insidious, gradually progressing to its current size of 3 x 3 cm and accompanied by pain. The patient also has a history of urinary hesitancy, burning micturition, and self-reported weight loss (measured by loosening of clothes). There was no noted history of nocturia, bowel complaints, fever, cough, and lower limb swelling. There were no associated comorbidities noted. Local examination showed a solitary ulceroproliferative growth of 3 x 3 cm over the penile prepuce extending up to the glans. The lump had an irregular surface and ill-defined margins without active bleeding or discharge. External meatus was normal. Penile edema was present, with no rise in local temperature. A 1 x 1 cm firm, smooth mobile lymph node palpable in the right inguinal region was observed with bilateral testis palpable (Figure 1).

**Figure 2:**
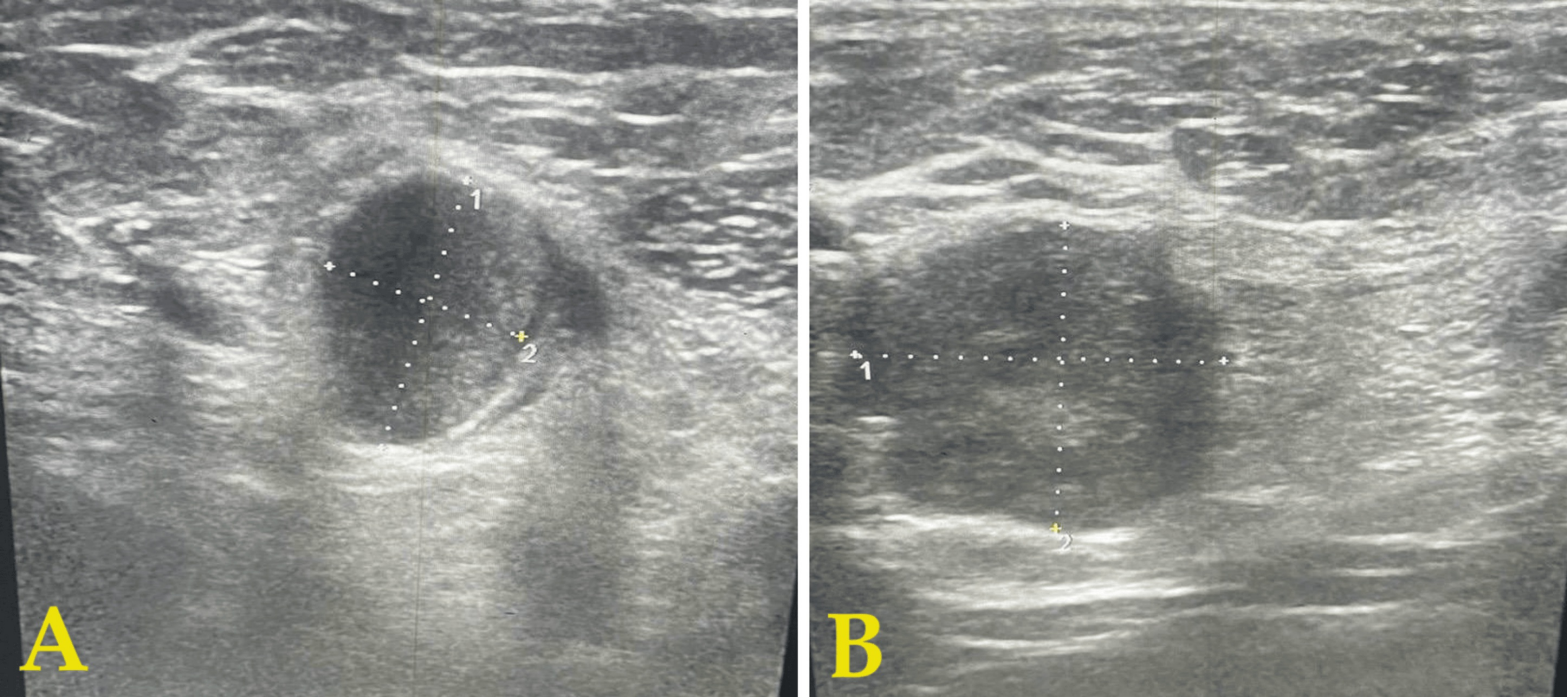
USG of the inguinoscrotal region suggestive of a right-sided inguinal hernia and right-sided edematous spermatic cord in (A) normal and (B) zoomed-in views USG: ultrasound imaging

Ultrasound imaging (USG) of the inguinoscrotal region was suggestive of a right-sided inguinal hernia and a right-sided edematous spermatic cord with raised vascularity, with a probable diagnosis of funiculitis, bilateral hydrocele (right > left), and inguinal lymphadenopathy (Figure 2).

**Figure 3:**
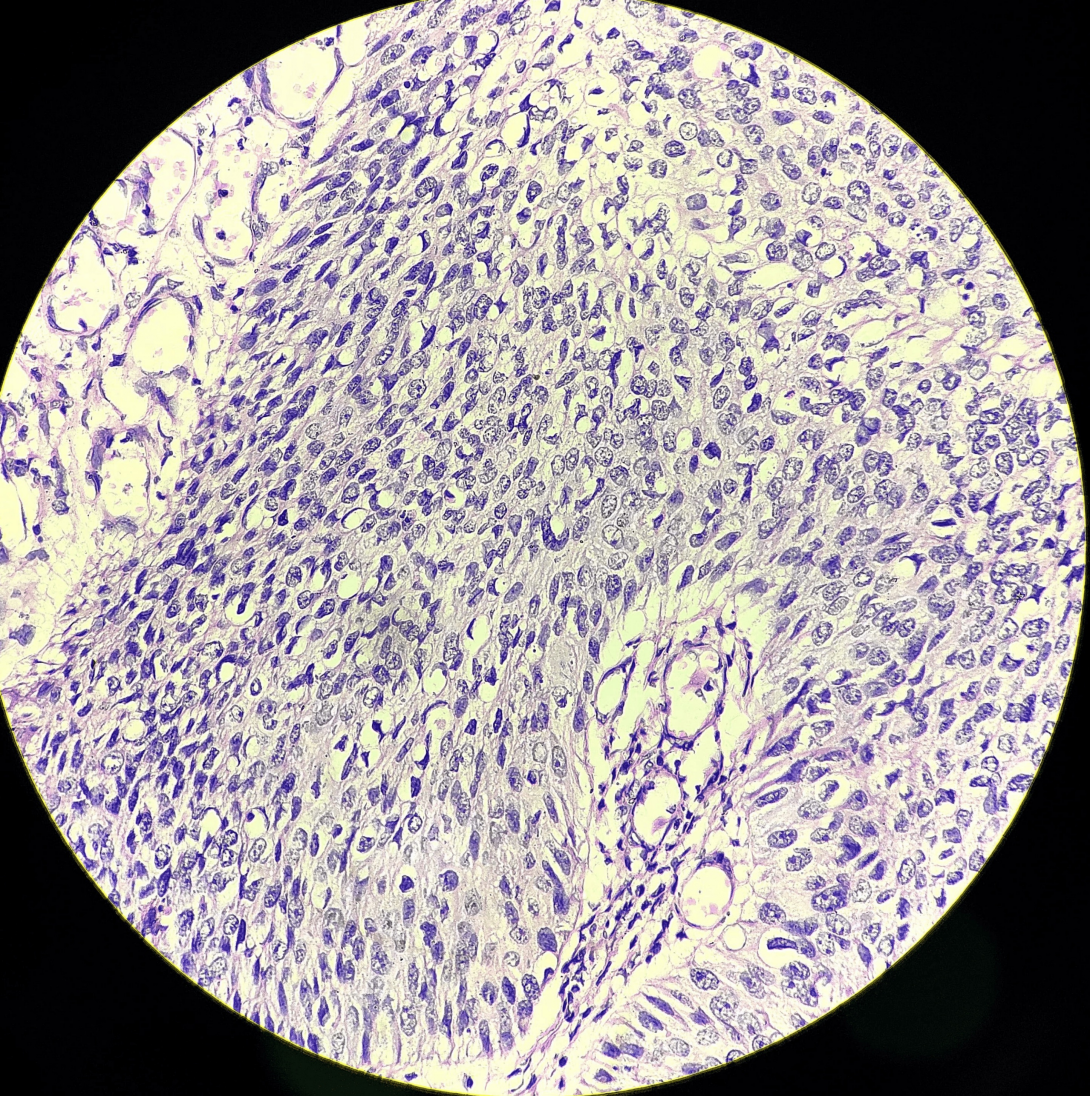
Hematoxylin and eosin-stained slide suggestive of penile intraepithelial neoplasia grade 3

USG of the abdomen pelvis was found to be normal. The patient was further subjected to wedge biopsy from the ulceroproliferative growth over prepuce. The histopathological examination of the same was suggestive of penile intraepithelial neoplasia grade 3 (Figure 3).

**Figure 4:**
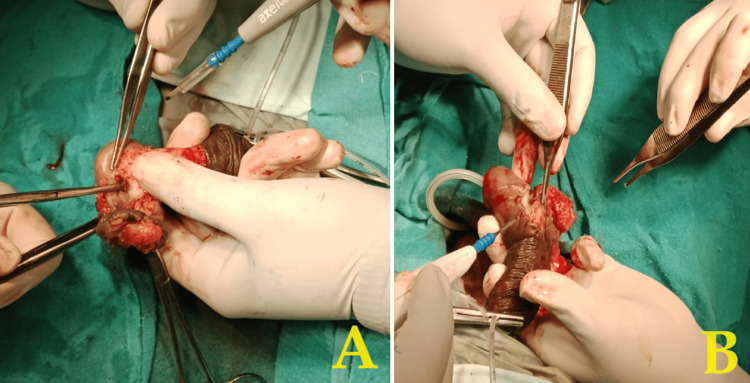
(A,B) Intraoperative image showing the dissection of growth from the base over the lateral aspect of penile region

**Figure 5:**
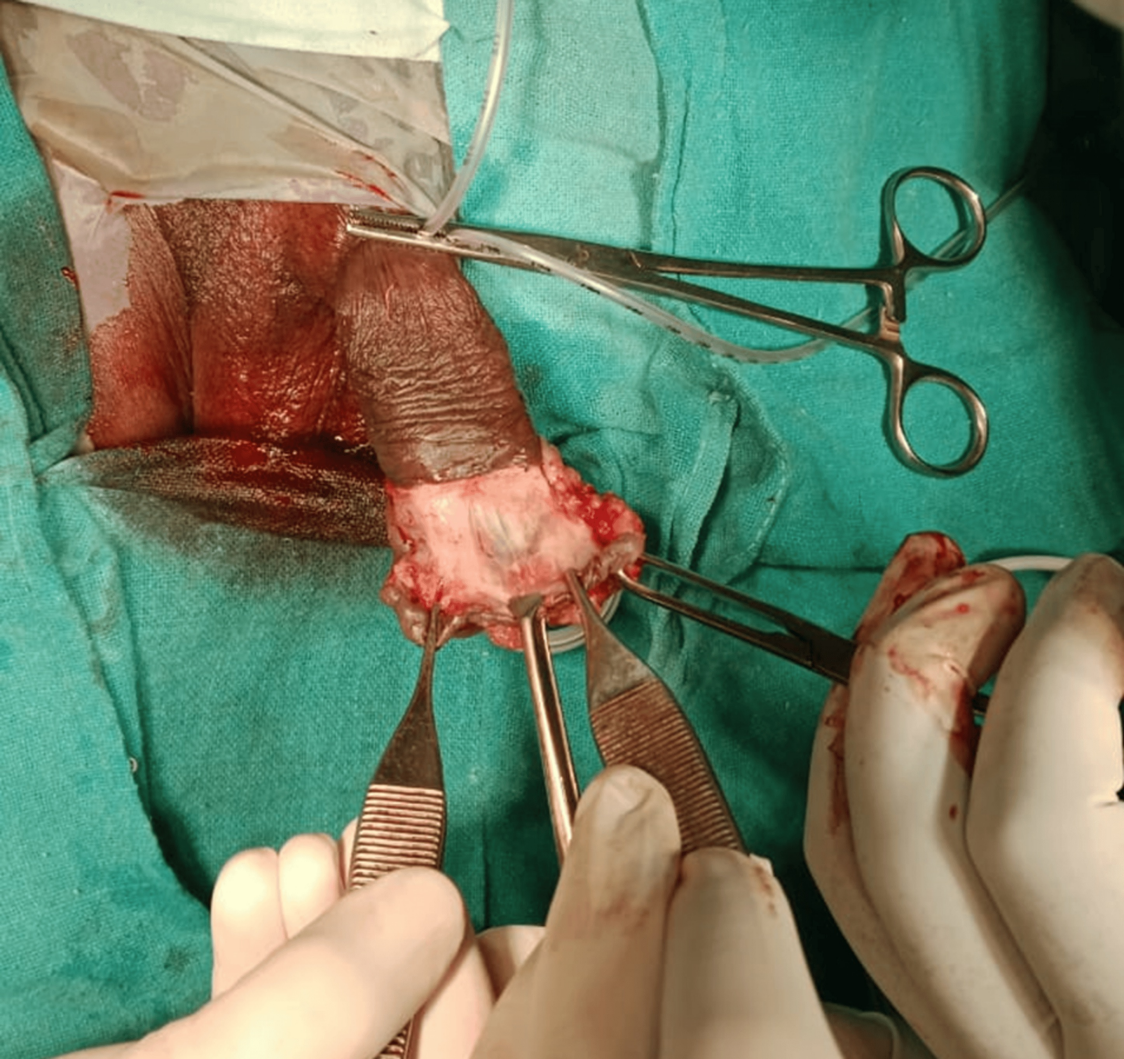
Intraoperative image showing the dissected plane of the growth from the superior margin

**Figure 6:**
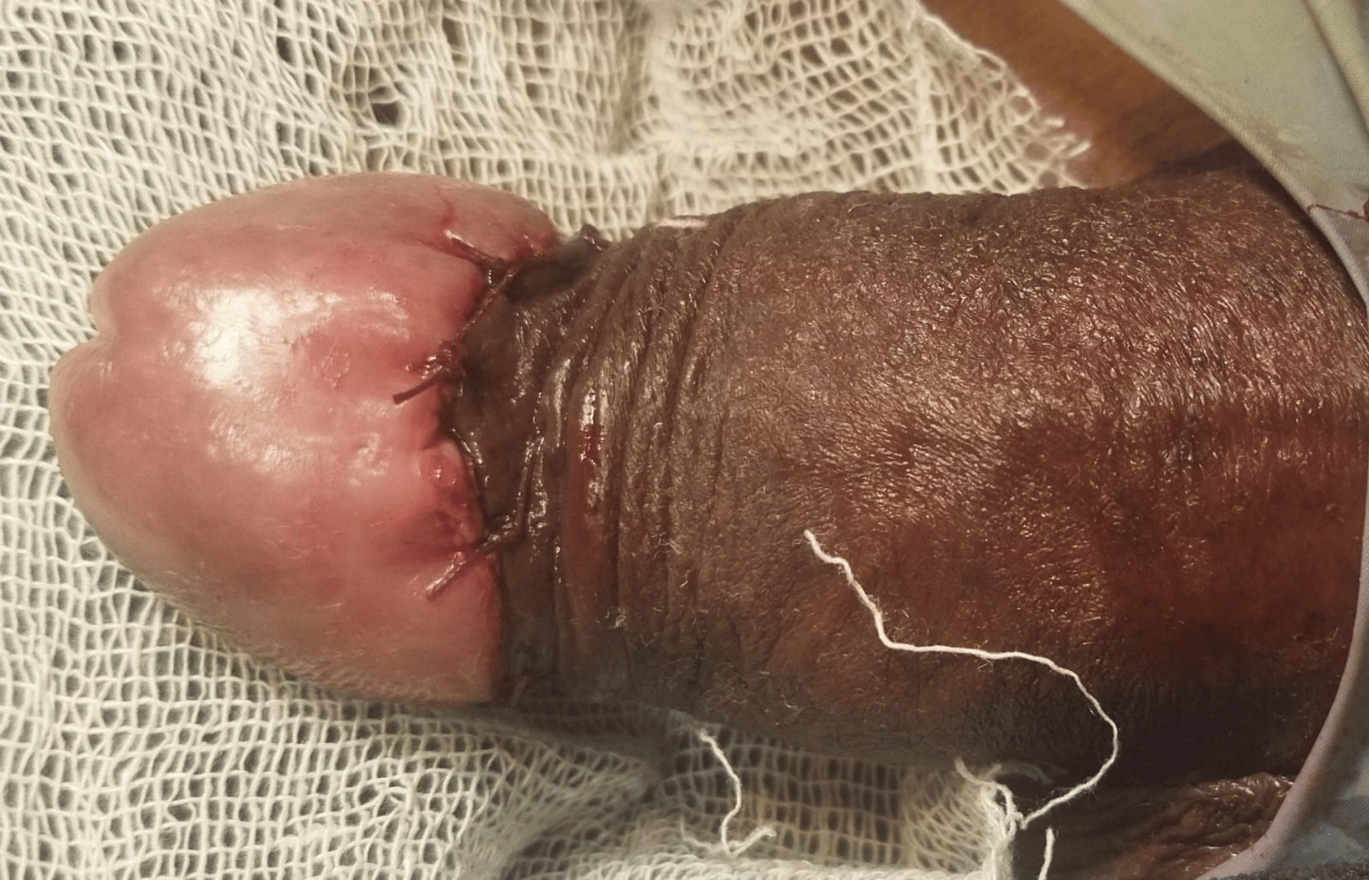
Postoperative image after penile reconstruction

Based on the final diagnosis of penile intraepithelial neoplasia grade 3, the patient was planned for wide local excision of penile tumor under spinal anesthesia. The excised specimen was sent for frozen section analysis, which was suggestive carcinoma in situ with negative margins. Hence, penectomy was not done, and only excision of growth was carried out with reconstruction for cosmetic satisfaction (Figures 4-6).

**Figure 7:**
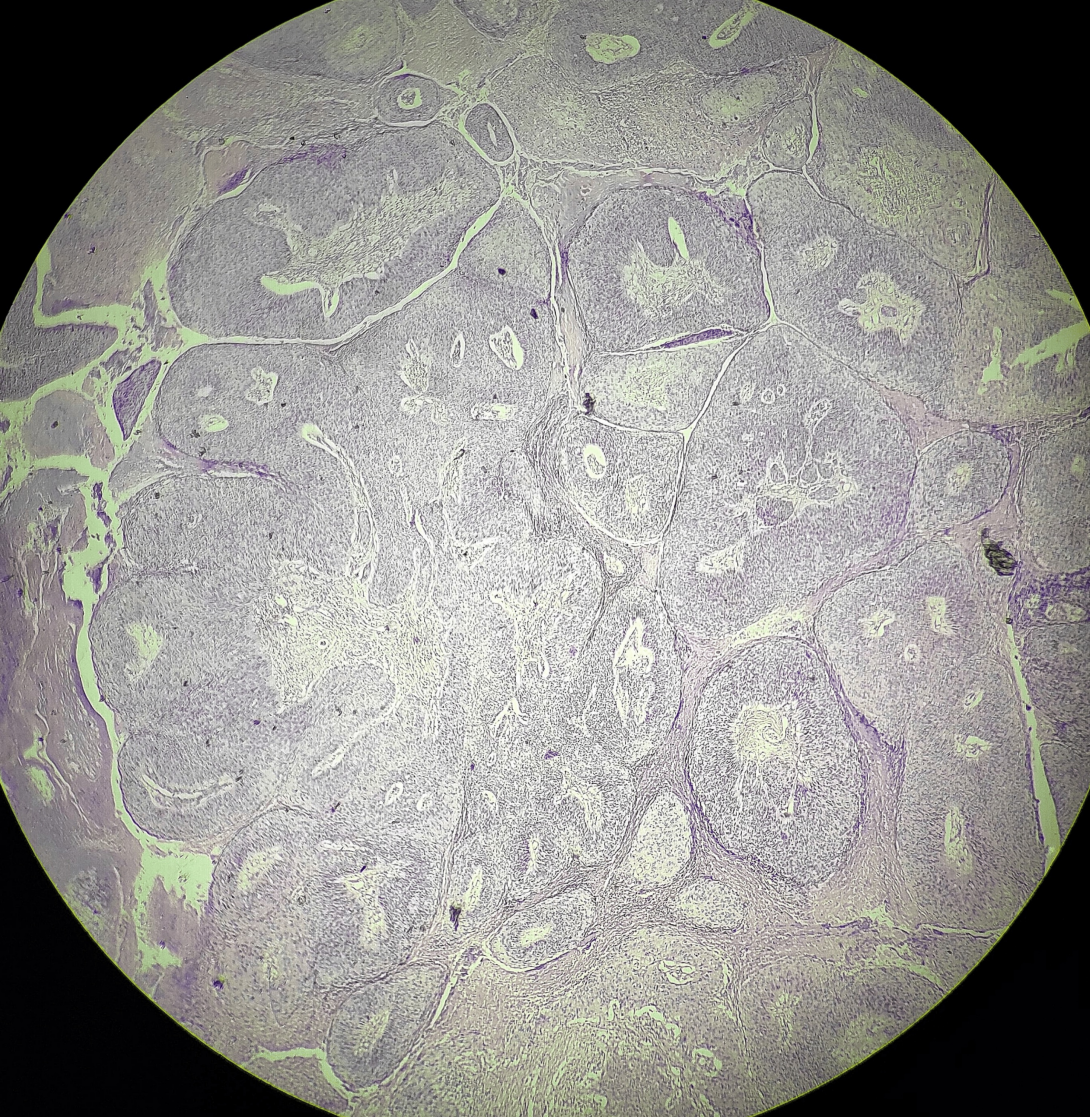
Hematoxylin and eosin-stained slide (10×) for histopathological examination suggestive of carcinoma in situ and negative margins

**Figure 8:**
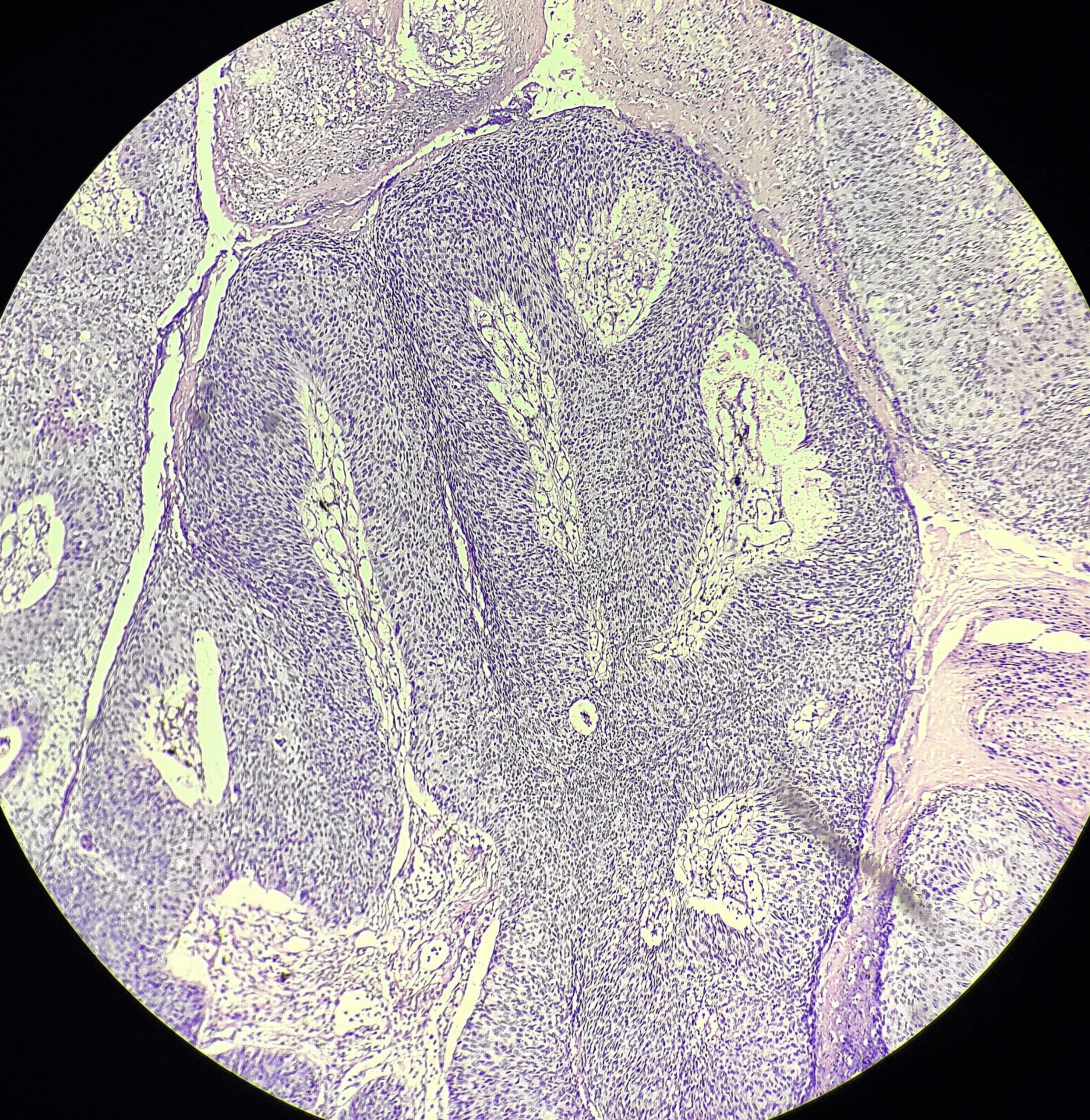
Hematoxylin and eosin-stained slide (40×) for histopathological examination suggestive of carcinoma in situ and negative margins

The excised specimen was sent for histopathological evaluation, which was suggestive of penile intraepithelial neoplasia grade 3, and margins were negative. The smears showed a well-distributed folliculated population of polymorphous lymphoid cells. It was composed of follicular center cells, small lymphocytes, and immunoblasts with few histiocytes and plasmacytoid lymphocytes without any malignant cells (Figures 7, 8).

On histopathological analysis, tissue sections from the proximal and distal margins were negative for infiltration by malignant cells. The patient came for follow-up after 15 days. The suture line was healthy. Further management was done as per the advice of a medical oncologist. The patient was advised to apply 5-fluorouracil locally for one month.

## Discussion

Penile intraepithelial neoplasia is rare in Asian males. Other locations for intraepithelial neoplasia are the neck, hands, trunk, oral cavity, head, buttocks, and nail bed, with a rare observation in the penis [5]. Penile intraepithelial neoplasia is a precancerous penile dermatosis where the epithelium displays dysplastic changes with an intact basement membrane. It displays atypical squamous cells with variations in size and shape, nuclear atypia, and increased mitotic activity. However, these changes are confined only to the epithelial layer compared to penile cancer, which is an invasive malignancy. Penile intraepithelial neoplasia may develop into cancer if not timely addressed [5,6]. Diagnosis can be carried out with the help of radiological assessment tools such as ultrasound, computed tomography, positron emission tomography, and magnetic resonance imaging along with physical presentation. However, a corresponding histopathological analysis is required to confirm the diagnosis [6]. Penile intraepithelial neoplasia was reported between 4% and 25% depending on the management strategies, viz. wide local incision, total glans resurfacing, glansectomy, and Mohs surgery [7]. Procedures that spare organs and glands can be used to treat low-risk cancers. If untreated, approximately 10%-30% of the penile intraepithelial neoplasia cases may progress to invasive squamous cell carcinoma, highlighting the importance of early detection and management [6,7]. Regular follow-up is essential due to the potential for recurrence after surgical treatment in 15%-20% of patients [8]. Though penile intraepithelial neoplasia is known to be common in males above 60 years of age, there are cases of age as young as 22 years male affected by the disease, which creates a need to emphasize the importance of reporting more cases [2,5]. Treatment of penile intraepithelial neoplasia generally requires surgical excision of the affected areas to prevent progression to invasive cancer and topical therapy, compared to penile cancers, which require a more vigorous multidisciplinary approach for its treatment [6,7]. Also, there is evidence of lower incidence of penile intraepithelial neoplasia in males undergoing circumcision. Though the underlying mechanisms are not clearly understood, it has been linked to lowered rates of human papillomavirus and other infections, reducing the chances of penile intraepithelial neoplasia [9]. This patient had a similar clinical presentation of a lesion with no urinary complaints, but unusual growth of the lesion. This clinical presentation was suggestive of malignancy, which was ruled out as a diagnosis of penile intraepithelial neoplasia grade 3 by histopathological analysis. Timely diagnosis and intervention can be counted as helpful in achieving a good outcome, maintaining penile function, achieving a satisfactory quality of life, and in the prevention of the development of invasive penile cancer.

## Conclusions

Though a rarity in Asian males with a common age of presentation of greater than 60 years, penile intraepithelial neoplasia is documented in younger males, too. Additional research must be carried out reporting clinical presentations and pathophysiology, which can aid early diagnosis and prompt treatment. This can provide crucial support in organ function preservation and significantly improve the physical and emotional quality of life of the patients.
